# The impact of multiple representations on students' understanding of vector field concepts: Implementation of simulations and sketching activities into lecture-based recitations in undergraduate physics

**DOI:** 10.3389/fpsyg.2022.1012787

**Published:** 2023-01-05

**Authors:** Larissa Hahn, Pascal Klein

**Affiliations:** Physics Education Research, Faculty of Physics, University of Göttingen, Göttingen, Germany

**Keywords:** multiple representations, simulation, conceptual understanding, vector fields, physics, sketching, task-based learning, lecture-based recitations

## Abstract

Multiple external representations (e.g., diagrams, equations) and their interpretations play a central role in science and science learning as research has shown that they can substantially facilitate the learning and understanding of science concepts. Therefore, multiple and particularly visual representations are a core element of university physics. In electrodynamics, which students encounter already at the beginning of their studies, vector fields are a central representation typically used in two forms: the algebraic representation as a formula and the visual representation depicted by a vector field diagram. While the former is valuable for quantitative calculations, vector field diagrams are beneficial for showing many properties of a field at a glance. However, benefiting from the mutual complementarity of both representations requires representational competencies aiming at referring different representations to each other. Yet, previous study results revealed several student problems particularly regarding the conceptual understanding of vector calculus concepts. Against this background, we have developed research-based, multi-representational learning tasks that focus on the visual interpretation of vector field diagrams aiming at enhancing a broad, mathematical as well as conceptual, understanding of vector calculus concepts. Following current trends in education research and considering cognitive psychology, the tasks incorporate sketching activities and interactive (computer-based) simulations to enhance multi-representational learning. In this article, we assess the impact of the learning tasks in a field study by implementing them into lecture-based recitations in a first-year electrodynamics course at the University of Göttingen. For this, a within- and between-subjects design is used comparing a multi-representational intervention group and a control group working on traditional calculation-based tasks. To analyze the impact of multiple representations, students' performance in a vector calculus test as well as their perceived cognitive load during task processing is compared between the groups. Moreover, analyses offer guidance for further design of multi-representational learning tasks in field-related physics topics.

## 1. Introduction

Mathematics and physics concepts are often represented in some form of external representation (De Cock, 2012). Thereby, different forms of representation, multiple representations (MRs), allow to express a concept or a (learning) subject in various manners by focusing on different properties and characteristics. In complementing and constraining each other, multiple representations enable a deep understanding of a situation or a construct (Ainsworth, 1999; Seufert, 2003) and, moreover, using multiple representations was found to have positive effects on knowledge acquisition and problem-solving skills (e.g., Nieminen et al., 2012; Rau, 2017). Regarding the understanding and communication of science concepts, visual representations are particularly crucial (Cook, 2006). Following previous research, they can help to eliminate science concepts' abstract nature and were shown to support students to develop scientific conceptions (e.g., Cook, 2006; Chiu and Linn, 2014; Suyatna et al., 2017). However, to benefit from multimedia learning environments, representational competencies based on an understanding of how individual representations depict information, how they relate to each other, and how to choose an appropriate representation to solve a problem are required (DeFT framework; Ainsworth, 2006). Without representational competencies, visual representations cannot fully unfold their potential as meaning-making tools.

Additionally, learning with and mentally processing visual representations often places special demands on the visuo-spatial working memory, thus increasing cognitive load (Baddeley, 1986; Cook, 2006; Logie, 2014). Here, previous research showed that externalizing visuo-spatial information can provide cognitive relief (e.g., Bilda and Gero, 2005). In this regard, sketching (or drawing) visual cues in multimedia learning has become an increasing scientific focus in recent years (Ainsworth and Scheiter, 2021). Following empirical findings, sketching allows to pay more attention to details (Ainsworth and Scheiter, 2021), thus supporting a visual understanding of concepts (Wu and Rau, 2018). Correspondingly, previous studies reported positive learning effects of sketching activities in (multi-)representational learning environments, as they increase attention and engagement with the representations and help to activate prior knowledge, to understand a representations' properties, or to recall information (e.g., Leopold and Leutner, 2012; Wu and Rau, 2018; Kohnle et al., 2020; Ainsworth and Scheiter, 2021). Typical sketching activities are copying a given representation, creating a visual representation with modified individual features or by transforming textual information into a drawing, or inventing a novel representation (e.g., to reason; Kohnle et al., 2020; Ainsworth and Scheiter, 2021). Moreover, with respect to Cognitive Load Theory (Sweller, 2010) which characterizes the limited capacity of working memory resources based on three types of cognitive load—intrinsic, extraneous, and germane cognitive load—sketching activities are able to promote a more effective use of these resources (Bilda and Gero, 2005). In addition to cognitive relief provided by sketching in multi-representational learning, previous work demonstrated the added value of interactive (computer-based) simulations for the development of representational competencies (e.g., Stieff, 2011; Kohnle and Passante, 2017). As such, integration of simulations in multimedia learning environments foster active learning, thus supporting students' use of scientific representations for communication and helping them to integrate their representational knowledge systematically with content knowledge (Linn et al., 2006; Stieff, 2011). Specifically, the complementation of simulation-based learning by the aforementioned sketching activities was found to support a deeper understanding of the representation being presented (Wu and Rau, 2018; Kohnle et al., 2020; Ainsworth and Scheiter, 2021).

Considering the value of multiple representations for science learning, unsurprisingly, they also play a major role in university physics. For instance, in electrodynamics, vector field representations are deeply rooted in the developmental history of the domain (Faraday, 1852), being represented either algebraically as a formula or graphically using arrows. In university experimental lectures, an introduction to electric and magnetic fields typically starts from concrete analogous representations of electric or magnetic field lines, then moving on to more abstract or idealized visual-graphical and symbolical representations (Küchemann et al., 2021). Using demonstration experiments, electric and magnetic field lines are visualized, for example, by semolina grains (Benimoff, 2006; Lincoln, 2017; Küchemann et al., 2021) or iron filings (Thompson, 1878; Küchemann et al., 2021), respectively. When representing a quantity as a vector field, the fields' properties, its divergence and curl, and further the integral theorems of Gauss and Stokes are of particular importance for physics applications (Griffiths, 2013). Accordingly, a sound understanding of vector calculus is of great importance for undergraduate and graduate physics studies. For example, a study by Burkholder et al. (2021) found a significant correlation between extensive preparation in vector calculus and students' performance in an introductory course on electromagnetism.

However, further research also revealed that a conceptual understanding, which is relevant to physics comprehension, often caused difficulties for students (e.g., Pepper et al., 2012; Singh and Maries, 2013; Bollen et al., 2015). Besides conceptual gaps regarding vector field representations in general, learning difficulties in dealing with vector field concepts such as divergence and curl became particularly apparent. For example, students struggled to extract information about divergence or curl from vector field diagrams and they interpreted and used these concepts literally instead of referring to their physics-mathematical concepts (Ambrose, 2004; Pepper et al., 2012; Singh and Maries, 2013; Bollen et al., 2015, 2016, 2018; Baily et al., 2016; Klein et al., 2018, 2019). In a study on students' difficulties regarding the curl of vector fields, Jung and Lee (2012) diagnosed the gap between mathematical and conceptual reasoning as a major source of comprehension problems. Furthermore, Singh and Maries (2013) concluded that graduate students struggle with the concepts of divergence and curl, even though they know how to calculate them mathematically. In the context of electrostatics and electromagnetism, it was also shown that conceptual gaps regarding vector calculus led to improper understanding and errors in when applying essential principles in physics of essential principles in physics (Ambrose, 2004; Jung and Lee, 2012; Bollen et al., 2015, 2016; Li and Singh, 2017). Regarding these findings, it is noticeable that the aforementioned studies did not strictly distinct between conceptual understanding and representational competencies with respect to vector fields. This is not surprising, since there is strong overlap of the two areas in this subject domain—vector fields are, as such, a form of representation that cannot be understood in a subject context isolated from concepts. Conversely, it is almost impossible to learn electrodynamics concepts without vector field representations.

In introductory physics texts, vector concepts are typically given as mathematical expressions, but are either not or insufficiently explained qualitatively (Smith, 2014). Even in more advanced physics textbooks, there is little geometric explanation or discussion of vector field concepts and integral theorems. Regarding the aforementioned empirical findings, relevance and requirement of new instructions that address a conceptual understanding become even more apparent. Consequently, numerous authors advocated the use of visual representations in order to foster a conceptual understanding. Following this line of research, Bollen et al. (2018) developed a guided-inquiry teaching-learning sequence on vector calculus in electrodynamics aiming at strengthening the connection between visual and algebraic representations. Implementing the tutorials in a second-year undergraduate electrodynamics course revealed a positive effect of the interventions on physics students' conceptual understanding and their ability to visually interpret vector field diagrams. In addition, subjects expressed primarily positive feedback regarding the learning approach. However, as discussed by the authors, the exact results should be interpreted with care as the number of participants was small and the implementation followed a less streamlined structure as, for example, no strict control and intervention group design was used. Additionally, Klein et al. (2018, 2019) developed text-based instructions for visually interpreting divergence using vector field diagrams. Eye tracking was used to analyze representation-specific visual behaviors, such as evaluating vectors along coordinate directions. Here, gaze analyses revealed a quantitative increase in conceptual understanding as a result of this intervention (Klein et al., 2018, 2019). In addition to a positive impact of visual cues on performance measures, a positive correlation with students' response confidence was found. This means that students not only answered correctly more often, but also trusted their answers more, which is a desirable result of successful teaching (Lindsey and Nagel, 2015; Klein et al., 2017, 2019). In subsequent interviews, subjects expressed diagram-specific mental operations, such as decomposing vectors and evaluating field components along coordinate directions, as a main problem source (Klein et al., 2018). Thus, a follow-up experimental study involved sketching activities aiming at generating representation-specific aids (e.g., field components) to support the visual interpretation of divergence (Hahn and Klein, 2021, 2022a). Here, sketching was shown to significantly reduce perceived cognitive load when applying visual problem-solving strategies related to a fields' divergence (Hahn and Klein, 2022a).

With regard to previous findings concerning student problems, building upon the existing multi-representational teaching-learning materials, and using the DeFT framework (Ainsworth, 2006), four multi-representational learning tasks were developed aiming at visually interpreting vector field diagrams (Hahn and Klein, 2022b). Their structure follows the Modeling Instruction approach as each task addresses one vector calculus concept in which the representational forms are used in a coordinated manner aiming at developing conceptual understanding (e.g., McPadden and Brewe, 2017). Furthermore, sketching activities and a vector field simulation are incorporated to provide cognitive relief, to foster engagement with the representations, and to support the development of representational competencies related to vector calculus concepts. Here, representation-specific sketching activities, such as sketching vector components or highlighting rows or columns to support evaluation along coordinate directions, were included (Klein et al., 2018, 2019; Hahn and Klein, 2021, 2022a). Additionally, typical sketching tasks for learning with simulations, such as copying or creating a vector field diagram, were involved (Kohnle et al., 2020). As part of the present registered report study, the research-based multi-representational learning tasks are implemented into lecture-based recitations in a first-year electrodynamics course. Consequently, the present study aims at evaluating the added value of multiple representations in task-based learning of vector calculus by comparing a multi-representational intervention group and a control group with traditional calculation-based task. Therefore, the following guiding question is investigated: “Do multi-representational learning tasks have a higher learning impact than traditional (calculation-based) tasks in the context of vector fields?” Considering previous research findings and theoretical frameworks from cognitive psychology on multi-representational learning, and on the use of sketching activities and simulations, we hypothesize that multi-representational, sketching- and simulation-based tasks

**(H1)** promote students' performance as measured by a vector field performance test (that includes tasks related to vector calculus, vector field quantities, and vector field concepts), and
**(H2)** reduce perceived cognitive load (as measured by a cognitive load questionnaire) during task processing.

## 2. Methods

Learning tasks are implemented in the weekly recitations on *experimental physics II* in the summer semester 2022 and 2023. Physics students usually attend *experimental physics II* in their second semester of study, then encountering university electromagnetism for the first time. The module includes a lecture with demonstration experiments and weekly recitations in which the compulsory assignments are discussed. Dividing the study into an alpha and a beta implementation (summer semester 2022 and 2023, respectively) primarily serves to consolidate the data. In the alpha implementation, all instruments and learning tasks are tested and psychometrically characterized, thus providing guidance for improvement. Then, alpha as well as beta implementation are used to evaluate the effectiveness of the intervention aiming at answering the guiding question and testing the hypotheses. Study design and procedure are identical in both implementations in order to transfer conclusions from the alpha to the beta implementation.

### 2.1. Procedure

**Figure 1:**
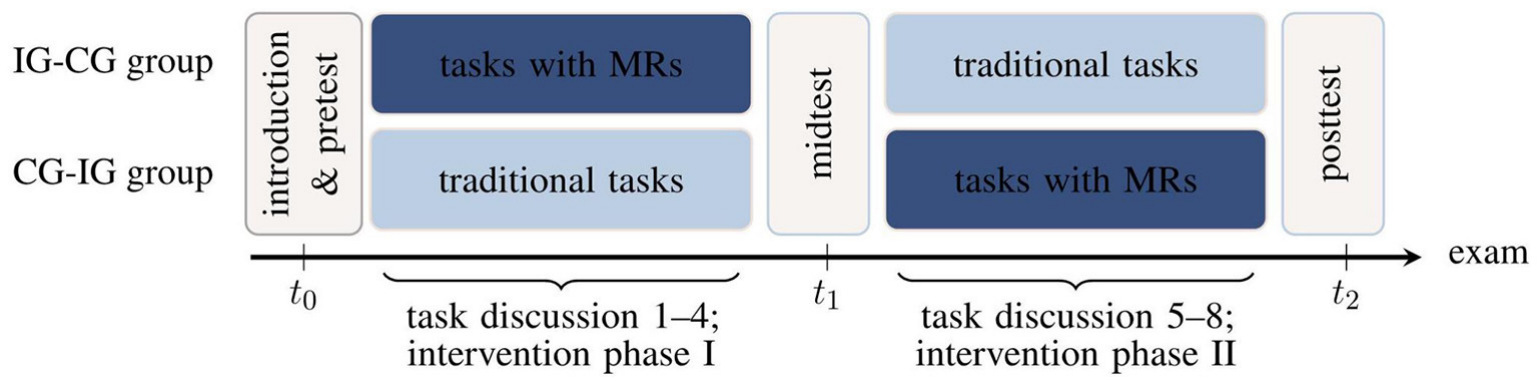
Study design with timeline from left (*t*_0_) to right (*t*_2_; intervention group IG, control group CG, multiple representations MRs). The designations “IG-CG group” and “CG-IG group” refer to the chronological order of the groups in the rotational design (first intervention group, then control group, or vice versa).

The studies are based on within- and between-subjects treatments wrapped in a rotational design (Figure 1). At the beginning of the lecture period, all recitation groups are randomly divided into two superordinate groups (IG-CG and CG-IG groups, respectively) both serving as intervention groups (IG) and control groups (CG) at some time but in different order. Students select a fixed recitation group by their own without knowing about the assignment to a treatment condition later on. Before the first intervention phase, students take a performance test on vector calculus (section 2.3). Subsequently, the first intervention phase starts and in each of the following four weeks, students complete a mandatory intervention task (either a multi-representational or a traditional task) in addition to a set of standard tasks which does not differ between the groups. The latter consists of typical, predominantly calculation- and formula-based, problem-solving tasks that have always been used in the course (e.g., they present some vector fields and students must calculate divergence or curl). First, the upper group in Figure 1 is intervention group (IG) and works on the multi-representational learning tasks, while the lower group, acting as a waiting group, is control group (CG) and works on traditional (calculation-based) tasks. All assignments are completed by self-study within one week, submitted for correction, and discussed with a dedicated, independent intervention tutor during the subsequent recitation. Prior to each task discussion, a short questionnaire on perceived cognitive load during task processing and means of task assistance is deployed (section 2.3). After the first intervention phase in the seventh week of the semester, students again complete the performance test on vector calculus and another evaluation questionnaire. Subsequently, the groups switch roles and the second four-week intervention phase starts. Finally, the performance test on vector calculus and the questionnaire are administered again.

### 2.2. Power analysis

Due to the lack of comparable studies regarding target group and topic, power analyses are based on effect sizes of methodologically similar studies. Akkus and Cakiroglu (2010) reported medium (η^2^ = 0.128, *f* = 0.383) to large effect sizes (η^2^ = 0.233, *f* = 0.551) when comparing seventh grade students' algebra performance between an experimental group provided with a multiple representation-based algebra instruction and a control group using a conventional instruction. Power analyses with G^⋆^Power 3.1 (Faul et al., 2009) indicated that for an analysis of covariance (*p* < 0.05) including two covariates (e.g., semester of study and school leaving examination grade) and an average effect size of *f* = 0.467, a sample size of *N* = 51 students would be required to obtain a desired power of 0.9 (McDonald, 2014). A meta analysis by Sokolowski (2018) found a large overall weighted mean effect size of *f* = 0.53 regarding the use of representations in Pre-K through fifth grade mathematics compared to traditional teaching methods which would require a sample size of *N* = 40 considering the aforementioned assumptions. Regarding the alpha implementation of this study, pre- and midtest were completed by *N* = 116 and *N* = 64 students, respectively. For beta implementation, similar sample sizes can be expected which would be consistent with the power analyses results.

### 2.3. Materials and measures

Test and scale analyses reported in the following are based on the assessments and responses of the alpha implementation. Here, data from the first implementation phase are used to ensure the largest possible data base. Therefore, the performance test at *t*_0_ (*N* = 116) and the weekly questionnaire used in the first recitation after the pretest (*N* = 93) were examined. The sample from the pretest included 86 male and 27 female undergraduate physics students with a mean age of 20.3 ± 1.9 years, a mean school leaving examination grade of 1.7 ± 0.6 [“Abitur” grade; referring to a scale from 1 (best) to 6], and in their 2.8 ± 1.5 semester of study. Prior to test and scale analyses, all datasets were cleaned of outliers. An overview of all variables and scales used in the study is given in Table 1.

**Table 1:** Overview of variables, instruments, and scales including scale analyses results of the alpha implementation (scale mean *M*, mean difficulty index *P*, mean standard deviation *SD*, mean discrimination index *D*, Cronbach's alpha α_*C*_, Spearman-Brown coefficient *ρ*).

**Variable**	**Scale**	**#item**	***M*/*P*^*a*^**	***SD***	***D***	**α_*C*_ /*ρ***
**Recommendations according to Ding and Beichner (** **2009** **)**		**–**	[0.3;0.9]^*b*^	**–**	≥ 0.3	≥ 0.7
**Dependent variables**						
Performance^*c*^	Vector calculus test (V)	65	0.51	0.13	0.34	0.86
Response confidence	Confidence (C)	42	0.52	0.23	0.56	0.97
Cognitive load	Extraneous cognitive load (ECL)	4	0.30	0.22	0.53	0.82
	Intrinsic cognitive load (ICL)	3	0.45	0.24	0.62	0.88
	Germane cognitive load (GCL)	2	0.57	0.25	0.63	0.84
	Effort (E)	2	0.67	0.25	0.63	0.80
**Independent variables**						
Group	–	–	–	–	–	–
Gender	–	–	–	–	–	–
**Control variables**						
Tutor behavior	Tutor (T)	6	0.84	0.14	0.33	0.89
Age	–	–	–	–	–	–
Semester of study	–	–	–	–	–	–
Abitur grade	–	–	–	–	–	–

a Scale mean is used for evaluation items; mean difficulty index is used for performance test items.

b Recommendation refers to mean difficulty index *P*.

c Multiple-choice and true-false items of one task are counted separately.

Initially, all subjects completed a test with demographic questions (e.g., age, gender, semester of study) and a performance test on vector calculus assessing conceptual understanding closely linked with representational competencies. The performance test included 19 tasks, partly comprising several subtasks, hence, a total of 65 items (multiple-choice and true-false items of one task counted separately) covering seven different subtopics of vector calculus. Forty-nine of the items were designed in multiple-choice or true-false format, while the remaining 16 items required a sketch, formula, justification, calculation, or a proof. Most of the items were taken from established concept tests on electrodynamics (CURrENT) or have been used and validated in a similar form in previous studies (Bollen et al., 2015, 2018; Baily et al., 2016, 2017; Klein et al., 2018, 2019, 2021; Hahn and Klein, 2022a; Rabe et al., 2022). Exploratory factor analysis of the performance test did not reveal a distinct factor structure which is a common result for concept tests in STEM education research (e.g., FCI; Heller and Huffman, 1995; Hestenes and Halloun, 1995; Huffman and Heller, 1995; Scott et al., 2012). Therefore, for the following analyses, the performance test is considered in its entirety. With a mean difficulty index of *P* = 0.51, a mean discrimination index of *D* = 0.34, and a reliability of α_*C*_ = 0.86 (Table 1), the performance test shows satisfactory psychometric properties according to the recommendations of Ding and Beichner (2009). Additionally, for most of the multiple-choice and true-false items, response confidence was assessed using a 6-point Likert-type rating scale (1 = absolutely confident to 6 = not confident at all; *D* = 0.56, α_*C*_ = 0.97; Table 1) to provide insight into student response behavior beyond performance measures. Since previous studies, also in the context of instruction-based learning of vector field concepts, found positive correlations between performance and confidence (e.g., Lindsey and Nagel, 2015; Klein et al., 2019), it will be investigated whether group membership influences this correlation.

In weekly recitations, students answered a short questionnaire related to the previous learning task providing information about the cognitive load they experienced while completing the task as well as any kind of task assistance. The items regarding cognitive load are based on a scale measuring the three types of cognitive load from Leppink et al. (2013) which was supplemented by items from Klepsch et al. (2017) and Krell (2017). The final questionnaire contained 12 items measuring cognitive load on a 6-point Likert-type rating scale (1 = strongly disagree to 6 = strongly agree). As a result of principal component analysis with varimax rotation $\left[\text{KMO}=0.74\text{,}X^{2}\left(66\right)=522.56\text{,}p<0.001\right]$, four subscales can be identified (75.03% variance explanation, excluding item CL10; Table 1): *extraneous cognitive load* (four items, α_*C*_ = 0.82), *intrinsic cognitive load* (three items, α_*C*_ = 0.88), *germane cognitive load* (two items, *ρ* = 0.84), and *effort* (two items, *ρ* = 0.80). The three scales for extraneous, intrinsic, and germane cognitive load reflect the three types of cognitive load according to Sweller (2010), with the germane cognitive load scale primarily addressing perceived improvement in understanding. In addition, the effort scale assesses the effort expended in task completion (Paas and Van Merriënboer, 1994; Krell, 2017). Following the recommendations of Ding and Beichner (2009), item and scale analyses yielded good values of item-total correlation (*r*_*it*_ ≥ 0.56) and discrimination indices (*D*_*i*_ ≥ 0.50) as well as the scales' mean discrimination indices (*D* ≥ 0.53) and their reliabilities (α_*C*_ ≥ 0.80). In addition to the perceived cognitive load, means of task assistance (e.g., “working together in a group with students from my course”, “looking up in a textbook”) were assessed using a choice format.

After the intervention phases, the students again completed the performance test which was extended by a module-specific task on electrostatics. In addition, a questionnaire was used which surveyed the tutor's behavior during task discussion as a control variable using six items (6-point Likert-type rating scale from 1 = strongly disagree to 6 = strongly agree). The items are based on the “tutor evaluation questionnaire” by Dolmans et al. (1994) supplemented by modifications from Baroffio et al. (1999) and Pinto et al. (2001). Following the results of principal component analysis with varimax rotation $\left[\text{KMO}=0.89\text{,}X^{2}\left(15\right)=293.91\text{,}p<0.001\right]$, the scale will be considered in its entirety (68.61% variance explanation). It shows a high reliability (α_*C*_ = 0.89) and a satisfactory discrimination index (*D* = 0.33; Table 1). Further information and detailed documentation of the study material, instruments, scales, and test analyses can be found in the Supplementary material.

### 2.4. Statistical data analysis

As required for parametric procedures, all scales for dependent and control variables (Table 1) were checked for normal distributed scale expressions. Regarding the hypotheses, statistical analyses will mainly comprise (co-)variance analyses to examine the influence of group membership on the dependent variables. Both intervention phases are methodologically treated equally, but analyzed separately to ensure the largest possible data base. Moreover, the rotational design allows a comparison of the pre-post learning gains of intervention and control group within each phase. Here, a common measure of gain, Hake's gain, as defined by the quotient of absolute gain and maximum possible gain, is used (Hake, 1998). However, a comparison of both conditions (traditional vs. multi-representational) can also take place within the IG-CG and CG-IG groups, since each group is once CG and once IG. In addition, 2 × 2 analyses of variance will be conducted to examine the impact of the intervention comparing different time points (pre-post comparison). Moreover, the performance test will be examined in more detail using Rasch analysis.

## Data availability statement

The raw data supporting the conclusions of this article will be made available by the authors, without undue reservation.

## Ethics statement

Ethical review and approval was not required for the study on human participants in accordance with the local legislation and institutional requirements. The patients/participants provided their written informed consent to participate in this study.

## Author contributions

PK supervised data collection and gave feedback to the first draft of the manuscript. LH performed the statistical analyses and wrote the first draft of the manuscript. All authors contributed to conception and design of the study.
