# Supplementary material for: The impact of multiple representations on students' understanding of vector field concepts: Implementation of simulations and sketching activities into lecture-based recitations in undergraduate physics
Source: Front Psychol. 2023 Jan 5;13:1012787. doi: 10.3389/fpsyg.2022.1012787 (PMC9849893; doi:10.3389/fpsyg.2022.1012787)
Supplement: Supplementary file 1 [file Data_Sheet_1.pdf]

Supplementary Material

The supplementary material follows the structure given below. For an impression of the performance test on vector calculus, the first half of the test is provided. Test and evaluation questionnaire items are presented in German original language; an English translations will be provided on request.

1 Study material
  - 1.1 Performance test on vector calculus
  - 1.2 Evaluation questionnaires

2 Test analyses
  - 2.1 Outlier analyses
  - 2.2 Factor analyses, item and scale analyses
    - 2.2.1 Performance test on vector calculus
    - 2.2.2 Evaluation questionnaires
  - 2.3 Normal distribution analyses

1 STUDY MATERIAL

1.1 Performance test on vector calculus

I Vektorfelder

V1) Gegeben ist das Schaubild des zweidimensionalen Vektorfeldes $\vec{F}(x, y)$. F_x bezeichnet die x-Komponente des Feldes $\vec{F}$ und F_y seine y-Komponente.

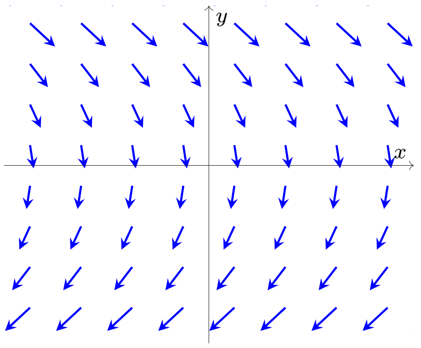

1. Kreuzen Sie an, welche der folgenden Formeln $\vec{F}(x, y)$ beschreibt.

☐ $\vec{F}_1(x, y) = x\hat{e}_x + y\hat{e}_y$

☐ $\vec{F}_2(x, y) = -\hat{e}_x + x\hat{e}_y$

☐ $\vec{F}_3(x, y) = y\hat{e}_x - \hat{e}_y$

☐ $\vec{F}_4(x, y) = y\hat{e}_x - x\hat{e}_y$

2. Kreuzen Sie an, ob die Aussage richtig oder falsch ist und geben Sie außerdem an, wie sicher Sie sich Ihrer Antwort sind (auf einer Skala von 1 = "absolut sicher" bis 6 = "absolut unsicher").

			**Antwortsicherheit**					
	**richtig**	**falsch**	**1**					**6**
**(a)** $F_x = 0$ überall	☐	☐	☐	☐	☐	☐	☐	☐
**(b)** $F_y = 0$ überall	☐	☐	☐	☐	☐	☐	☐	☐

V2) Gegeben ist das Vektorfeld $\vec{G}(r, \varphi) = r\hat{e}_\varphi$.

1. Skizzieren Sie $\vec{G}(r, \varphi)$ in der x-y-Ebene.

2. Kreuzen Sie an, ob die Aussage richtig oder falsch ist und geben Sie außerdem an, wie sicher Sie sich Ihrer Antwort sind (auf einer Skala von 1 = "absolut sicher" bis 6 = "absolut unsicher").

			**Antwortsicherheit**					
	**richtig**	**falsch**	**1**					**6**
**(a)** $\vec{G}$ besitzt nur eine r-Komponente, die von φ abhängt.	☐	☐	☐	☐	☐	☐	☐	☐
**(b)** $\vec{G}$ besitzt nur eine φ-Komponente, die von r abhängt.	☐	☐	☐	☐	☐	☐	☐	☐

II Divergenz von Vektorfeldern

V3) Geben Sie einen mathematischen Ausdruck (eine Formel) zur Berechnung der Divergenz eines Vektorfeldes $\vec{F}(x, y)$ in kartesischen Koordinaten an.

☐ Mir fällt gerade keine Formel ein.

V4) Gegeben ist das folgende Schaubild eines Vektorfeldes $\vec{F}(x, y)$.

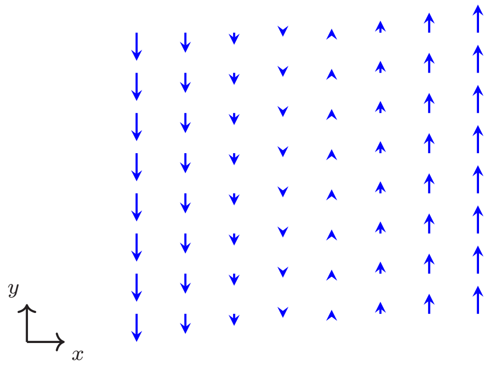

1. Welche Aussage können Sie über die Divergenz des Vektorfeldes formulieren? Markieren Sie Ihre Antwort!

☐ Das Vektorfeld besitzt keine Divergenz. (Das bedeutet, dass die Divergenz an jeder Stelle gleich Null ist.)

☐ Die Divergenz ist an wenigstens einer Stelle im Vektorfeld von Null verschieden.

☐ Die Divergenz ist an jeder Stelle im Vektorfeld von Null verschieden.

2. Begründen Sie Ihre Antwort kurz.

V5) Kreuzen Sie an, ob die folgenden Aussagen über die Divergenz richtig bzw. falsch sind und geben Sie außerdem an, wie sicher Sie sich Ihrer Antwort sind (auf einer Skala von 1 = "absolut sicher" bis 6 = "absolut unsicher").

			**Antwortsicherheit**					
	**richtig**	**falsch**	**1**					**6**
**1.** Die Divergenz gibt die Richtung des steilsten Anstiegs an.	☐	☐	☐	☐	☐	☐	☐	☐
**2.** Wenn die Divergenz eines Vektorfeldes an jeder Stelle positiv ist, existiert an jeder Stelle eine Quelle.	☐	☐	☐	☐	☐	☐	☐	☐

			Antwortsicherheit					
	**richtig**	**falsch**	**1**					**6**
**3.** Die Divergenz gibt an, ob die Vektorpfeile auseinanderlaufen.	☐	☐	☐	☐	☐	☐	☐	☐
**4.** Die Divergenz kann für jede Stelle eines Vektorfeldes verschieden sein.	☐	☐	☐	☐	☐	☐	☐	☐
**5.** Die Divergenz ist eine globale Feldeigenschaft: Entweder sie ist räumlich konstant oder Null.	☐	☐	☐	☐	☐	☐	☐	☐
**6.** Die Divergenz kann mit der Anzahl von Vektoren in Verbindung gebracht werden.	☐	☐	☐	☐	☐	☐	☐	☐
**7.** Die Divergenz hängt mit infinitisimalen Feldänderungen zusammen.	☐	☐	☐	☐	☐	☐	☐	☐
**8.** Das Feld einer Punktladung in der Umgebung dieser Ladung ist divergenzfrei.	☐	☐	☐	☐	☐	☐	☐	☐
**9.** Für die Divergenz gilt: div $\vec{F}$ = ...<br>☐ $\nabla\vec{F}$<br>☐ $\nabla\times\vec{F}$<br>☐ $\nabla\cdot\vec{F}$			☐	☐	☐	☐	☐	☐
**10.** Die Divergenz an einer Stelle eines Vektorfeldes ist ...<br>☐ ein Skalar<br>☐ ein Skalarfeld<br>☐ ein Vektor<br>☐ ein Vektorfeld			☐	☐	☐	☐	☐	☐
**11.** Die Divergenz des Vektorfeldes $\vec{F}(r,\varphi)$ in ebenen Polarkoordinaten ist definiert durch ...<br>☐ div $\vec{F} = \frac{\partial}{\partial r}F_r + \frac{\partial}{\partial\varphi}F_\varphi$<br>☐ div $\vec{F} = \frac{\partial}{\partial r}(rF_r) + \frac{\partial}{\partial\varphi}F_\varphi$<br>☐ div $\vec{F} = \frac{1}{r}\frac{\partial}{\partial r}(rF_r) + \frac{1}{r}\frac{\partial}{\partial\varphi}F_\varphi$<br>☐ div $\vec{F} = \frac{1}{r}\frac{\partial}{\partial r}(rF_r) + \frac{1}{r}\frac{\partial}{\partial\varphi}(r^2F_\varphi)$			☐	☐	☐	☐	☐	☐

V6) Berechnen Sie die Divergenz des Vektorfeldes $\vec{F}(x,y,z) = x\hat{e}_x + x\hat{e}_y - 2xz\hat{e}_z$.

III Rotation von Vektorfeldern

V7) Geben Sie einen mathematischen Ausdruck (eine Formel) zur Berechnung der Rotation eines Vektorfeldes $\vec{F}(x,y,z)$ in kartesischen Koordinaten an.

____

☐ Mir fällt gerade keine Formel ein.

V8) Gegeben ist das folgende Schaubild eines Vektorfeldes $\vec{F}(x,y)$.

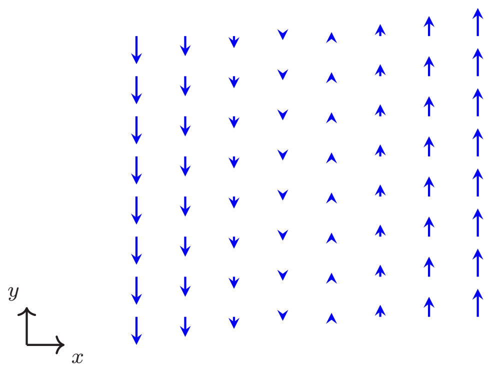

1. Welche Aussage können Sie über die Rotation des Vektorfeldes formulieren? Markieren Sie Ihre Antwort!

☐ Das Vektorfeld besitzt keine Rotation. (Das bedeutet, dass die Rotation an jeder Stelle gleich Null ist.)

☐ Die Rotation ist an wenigstens einer Stelle im Vektorfeld von Null verschieden.

☐ Die Rotation ist an jeder Stelle im Vektorfeld von Null verschieden.

2. Begründen Sie Ihre Antwort kurz.

____

____

V9) Kreuzen Sie an, ob die folgenden Aussagen über die Rotation richtig bzw. falsch sind und geben Sie außerdem an, wie sicher Sie sich Ihrer Antwort sind (auf einer Skala von 1 = "absolut sicher" bis 6 = "absolut unsicher").

			Antwortsicherheit					
	**richtig**	**falsch**	**1**					**6**
**1.** Die Rotation gibt die Richtung des steilsten Anstiegs an.	☐	☐	☐	☐	☐	☐	☐	☐
**2.** Die Rotation ist nur dann verschieden von Null, wenn sich die Richtung des Feldes ändert.	☐	☐	☐	☐	☐	☐	☐	☐
**3.** Die Rotation gibt an, ob die Vektorpfeile zusammenlaufen.	☐	☐	☐	☐	☐	☐	☐	☐
**4.** Die Rotation kann für jede Stelle eines Vektorfeldes verschieden sein.	☐	☐	☐	☐	☐	☐	☐	☐

	richtig	falsch	Antwortsicherheit 1					6
**5.** Die Rotation ist eine globale Feldeigenschaft: Entweder sie ist räumlich konstant oder Null.	☐	☐	☐	☐	☐	☐	☐	☐
**6.** Die Rotation gibt an, ob Feldvektoren gekrümmt sind.	☐	☐	☐	☐	☐	☐	☐	☐
**7.** Die Rotation ist ein Maß für infinitisimale Wirbel eines Feldes.	☐	☐	☐	☐	☐	☐	☐	☐
**8.** Das Feld einer Punktladung in der Umgebung dieser Ladung ist rotationsfrei.	☐	☐	☐	☐	☐	☐	☐	☐
**9.** Für die Rotation gilt: rot $\vec{F}$ = ... ☐ $\nabla\vec{F}$ ☐ $\nabla\times\vec{F}$ ☐ $\nabla\cdot\vec{F}$			☐	☐	☐	☐	☐	☐
**10.** Die Rotation an einer Stelle eines Vektorfeldes ist ... ☐ ein Skalar ☐ ein Skalarfeld ☐ ein Vektor ☐ ein Vektorfeld			☐	☐	☐	☐	☐	☐
**11.** Die Rotation des Vektorfeldes $\vec{F}(r,\varphi,z=0)$ in Zylinderkoordinaten ist definiert durch ... ☐ rot $\vec{F}=\left(\frac{\partial F_\varphi}{\partial r}-\frac{\partial F_r}{\partial\varphi}\right)\hat{e}_z$ ☐ rot $\vec{F}=\left(\frac{\partial}{\partial r}(rF_\varphi)-\frac{\partial F_r}{\partial\varphi}\right)\hat{e}_z$ ☐ rot $\vec{F}=\frac{1}{r}\left(\frac{\partial}{\partial r}(rF_\varphi)-\frac{\partial F_r}{\partial\varphi}\right)\hat{e}_z$ ☐ rot $\vec{F}=\frac{1}{r}\left(\frac{\partial}{\partial r}(rF_\varphi)-\frac{\partial}{\partial\varphi}(r^2F_r)\right)\hat{e}_z$			☐	☐	☐	☐	☐	☐

V10) Berechnen Sie die Rotation des Vektorfeldes $\vec{F}(x,y,z)=x\hat{e}_x+x\hat{e}_y-2xz\hat{e}_z$.

IV Partielle Ableitungen

V11) Gegeben sind die Schaubilder der zweidimensionalen Vektorfelder $\vec{F}(x,y)$ und $\vec{G}(x,y)$. F_x bezeichnet die x-Komponente des Feldes $\vec{F}$ (analog für alle weiteren Komponenten).

$\vec{F}(x,y)$

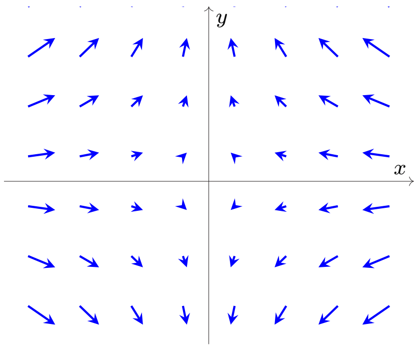

$\vec{G}(x,y)$

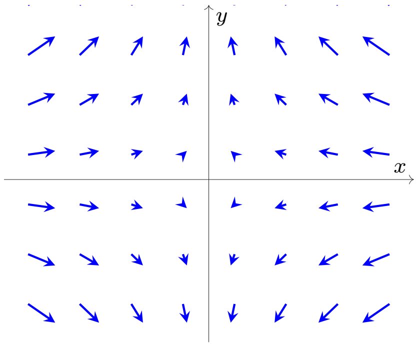

Markieren Sie, welches der Vergleichszeichen (>0, <0 oder $=0$) für die partiellen Ableitungen $\frac{\partial F_x}{\partial x}$, $\frac{\partial F_y}{\partial y}$, $\frac{\partial F_x}{\partial y}$ und $\frac{\partial F_y}{\partial x}$ (analog für $\vec{G}$) von $\vec{F}$ und $\vec{G}$ gilt. Geben Sie außerdem an, wie sicher Sie sich Ihrer Antwort sind (auf einer Skala von 1 = " absolut sicher" bis 6 = "absolut unsicher").

	>0	<0	$=0$	Antwortsicherheit 1					6
**1.** $\frac{\partial F_x}{\partial x}$	☐	☐	☐	☐	☐	☐	☐	☐	☐
**2.** $\frac{\partial F_y}{\partial y}$	☐	☐	☐	☐	☐	☐	☐	☐	☐
**3.** $\frac{\partial F_x}{\partial y}$	☐	☐	☐	☐	☐	☐	☐	☐	☐
**4.** $\frac{\partial F_y}{\partial x}$	☐	☐	☐	☐	☐	☐	☐	☐	☐
**5.** $\frac{\partial G_x}{\partial x}$	☐	☐	☐	☐	☐	☐	☐	☐	☐
**6.** $\frac{\partial G_y}{\partial y}$	☐	☐	☐	☐	☐	☐	☐	☐	☐
**7.** $\frac{\partial G_x}{\partial y}$	☐	☐	☐	☐	☐	☐	☐	☐	☐
**8.** $\frac{\partial G_y}{\partial x}$	☐	☐	☐	☐	☐	☐	☐	☐	☐

Table S1. Overview of topic, task type, and source of performance test tasks (topics: vector fields VF, divergence DIV, curl CU, partial derivatives PD; task type: multiple-choice item with three/four options MC3/4, true-false item TF, Sketch S, Open format O (formula, justification, or calculation)). Tasks that ask for response confidence are marked with * (asterisks).

Task	Topic	Task type	Source
V1	VF	MC4, TF*	Klein et al. (2018, 2019, 2021)
V2	VF	S, TF*	–
V3	DIV	O	Bollen et al. (2015); Klein et al. (2018, 2019, 2021)
V4	DIV	MC3, O	Klein et al. (2018, 2019, 2021)
V5	DIV	TF*, MC3*, MC4*	Baily et al. (2016); Bollen et al. (2018); Hahn and Klein (2022); Klein et al. (2018, 2019, 2021)
V6	DIV	O	Bollen et al. (2015)
V7	CU	O	Klein et al. (2018, 2019, 2021)
V8	CU	MC3, O	Bollen et al. (2015)
V9	CU	TF*, MC3*, MC4*	Baily et al. (2016); Bollen et al. (2018); Hahn and Klein (2022); Klein et al. (2018, 2019, 2021)
V10	CU	O	Bollen et al. (2015)
V11	PD	MC3*	Hahn and Klein (2022)

1.2 Evaluation questionnaires

Table S2: Questionnaire items with sources (cognitive load items CL, tutor items T).

Item	Item phrasing	Source
Cognitive load (CL)[a]		
CL1	Das behandelte Thema der Divergenz von Vektorfeldern habe ich als komplex empfunden.	LPC1
CL2	Die behandelten Formeln zur Divergenz habe ich als komplex empfunden.	LPC2
CL3	Die behandelten Konzepte und Definitionen zur Divergenz habe ich als komplex empfunden.	LPC3
CL4	Die Aufgabenstellungen waren unklar.	LPC4
CL5	In den Aufgabenstellungen wurden unklare und unbekannte Begriffe verwendet.	LPC6
CL6	Bei dieser Aufgabe war es mühsam, die wichtigsten Informationen zu erkennen.	KSS6
CL7	Bei dieser Aufgabe war es schwer, die zentralen Inhalte miteinander in Verbindung zu bringen.	KSS8
CL8	Die Aufgabe enthielt Elemente, die mich unterstützten, das Divergenzkonzept besser zu verstehen.	KSS7
CL9	Die Aufgabe hat mein Verständnis der Divergenz verbessert.	LPC7
CL10	Die Aufgabe hat mein Verständnis der Formeln zur Divergenz verbessert.	LPC9
CL11	Ich habe mir Mühe gegeben, um diese Aufgabe zu lösen.	KRE12
CL12	Es ging mir beim Bearbeiten der Aufgabe darum, alles richtig zu verstehen.	KSS4
Tutor (T)[b]		
T1	Die Tutorin wirkte motiviert.	DWS11
T2	Die Tutorin war ansprechbar für Fragen.	BKV9/10

T3	Die Tutorin motivierte zur aktiven Mitarbeit aller Teilnehmenden.	DWS2
T4	Die Tutorin zeigte Interesse am Lernerfolg der Studierenden.	DWS12
T5	Die Tutorin trug zu einem besseren Verständnis der Übungsaufgaben bei.	DWS10
T6	Die Tutorin wirkte gut vorbereitet.	DWS1

[a]Source abbreviations CL: LPC (Leppink et al., 2013), KRE (Krell, 2017), KSS (Klepsch et al., 2017)
[b]Source abbreviations T: DWS (Dolmans et al., 1994) BKV (Baroffio et al., 1999)

2 TEST ANALYSES

Since all data except the performance test data were given in values between 1 and 6, a linear transformation to the interval $[0; 1]$ was performed. Then, in order to form content-valid and psychometrically reliable scales, questionnaires were subjected to test analyses. For this purpose, the collected data were first checked for outliers before subjecting the outlier-corrected data to explorative factor analyses. Subsequently, item and scale analyses were conducted and the scales' psychometric parameters were determined. Finally, all scales were checked for normal distributed scale expressions. Analyses were performed with SPSS (version 27), Excel, or R (version 3.6.3, packages *foreign* and *polycor*).

2.1 Outlier analyses

Initially, all questionnaires except the performance test were subjected to outlier analyses. Number and frequency of data points whose $|z|$ values exceed the critical value of 3.29 (Tabachnick and Fidell, 2019) are shown in Table S3. Outliers were winsorized at the 99.9th percentile ($M \pm 3SD$; sign determined by outlier direction).

Table S3. Item-level outlier analyses.

Questionnaire	Outlier $|z| > 3.29$	Outlier [%]
Confidence	0	0
Cognitive load	0	0
Tutor behavior	13	2.55

2.2 Factor analyses, item and scale analyses

2.2.1 Performance test on vector calculus

For analyzing the performance test, a core item group of items which were included in pre-, mid-, and posttest were selected. For 65 dichotomous core items, tetrachoric correlations were determined and factor analysis was performed (Scott et al., 2012). However, as the matrix was singular, it was not appropriate for factor analysis. Results of item analyses (Table S4) showed that the guidelines for difficulty index, discrimination index, and item-total correlation according to Ding and Beichner (2009) were not fulfilled for most of the items. Therefore, after reducing the number of items based on these criteria, factor analysis based on the tetrachoric matrix was repeated. However, no clear factor structure was found. Therefore, the test is considered in its entirety (Table S4, scale analysis). Further analyses (e.g. Rasch analysis) are in prospect. Since an exploratory factor analysis of the response confidence did not yield a clear factor structure neither, this scale is also considered in its entirety (Table S5).

Table S4: Results of item and scale analyses of the performance test (item difficulty index P_i, item discrimination index D_i, item-total correlation r_{it}, Crombach's alpha when item i is excluded α_{ex}, mean difficulty index P, mean discrimination index D, standard deviation SD, Crombach's Alpha α_C).

	Item analysis			
Item	P_i	D_i	r_{it}	α_{ex}
V1.1	0.61	0.59	0.45	0.85
V1.2a	0.90	0.28	0.32	0.85
V1.2b	0.95	0.14	0.29	0.85
V2.1	0.52	0.52	0.38	0.85
V2.2a	0.86	0.24	0.27	0.85
V2.2b	0.73	0.31	0.24	0.85
V3	0.44	0.66	0.47	0.85
V4.1	0.39	0.48	0.35	0.85
V4.2	0.20	0.55	0.54	0.85
V5.1	0.64	0.17	0.12	0.86
V5.2	0.52	0.03	0.01	0.86
V5.3	0.36	-0.17	-0.23	0.86
V5.4	0.77	0.31	0.18	0.86
V5.5	0.82	0.31	0.30	0.85
V5.6	0.70	0.31	0.12	0.86
V5.7	0.83	0.24	0.20	0.85
V5.8	0.25	-0.10	-0.18	0.86
V5.9	0.73	0.34	0.22	0.85
V5.10	0.71	0.28	0.20	0.86
V5.11	0.41	0.59	0.40	0.85
V6	0.43	0.66	0.42	0.85
V7	0.30	0.41	0.29	0.85
V8.1	0.17	0.24	0.23	0.85
V8.2	0.14	0.28	0.28	0.85
V9.1	0.90	0.31	0.35	0.85
V9.2	0.28	0.21	0.14	0.86
V9.3	0.80	0.28	0.28	0.85
V9.4	0.69	0.55	0.39	0.85
V9.5	0.68	0.59	0.45	0.85
V9.6	0.47	0.34	0.23	0.85
V9.7	0.89	0.14	0.16	0.86
V9.8	0.73	0.59	0.44	0.85
V9.9	0.92	0.28	0.30	0.85
V9.10	0.63	0.45	0.26	0.85
V9.11	0.32	0.31	0.20	0.86
V10	0.34	0.69	0.45	0.85
V11.1	0.68	0.45	0.36	0.85
V11.2	0.84	0.34	0.32	0.85
V11.3	0.52	0.69	0.47	0.85
V11.4	0.54	0.59	0.44	0.85

V11.5	0.72	0.55	0.43	0.85
V11.6	0.82	0.45	0.36	0.85
V11.7	0.66	0.55	0.47	0.85
V11.8	0.72	0.31	0.31	0.85
...				
Scale	P	SD	D	α_C
Vector calculus test	0.51	0.13	0.34	0.86

Table S5: Item and scale analyses results of response confidence in the performance test (item mean M_i, item standard deviation SD_i, item discrimination index D_i, item-total correlation r_{it}, Crombach's alpha when item i is excluded α_{ex}, scale mean M, mean standard deviation SD, mean discrimination index D, Crombach's Alpha α_C).

	Item analysis				
Item	M_i	SD_i	D_i	r_{it}	α_{ex}
V1.2a	0.44	0.38	0.44	0.46	0.97
V1.2b	0.43	0.37	0.44	0.47	0.96
V2.2a	0.44	0.35	0.40	0.44	0.97
V2.2b	0.44	0.36	0.47	0.47	0.97
V5.1	0.49	0.31	0.49	0.61	0.96
V5.2	0.53	0.36	0.53	0.54	0.96
V5.3	0.53	0.34	0.65	0.71	0.96
V5.4	0.45	0.29	0.40	0.53	0.96
V5.5	0.46	0.38	0.60	0.61	0.96
V5.6	0.50	0.35	0.51	0.54	0.96
V5.7	0.41	0.34	0.55	0.68	0.96
V5.8	0.54	0.37	0.75	0.80	0.96
V5.9	0.37	0.38	0.42	0.40	0.97
V5.10	0.43	0.34	0.45	0.54	0.96
V5.11	0.55	0.36	0.40	0.42	0.97
V9.1	0.54	0.36	0.51	0.51	0.96
V9.2	0.57	0.33	0.53	0.64	0.96
V9.3	0.55	0.34	0.49	0.59	0.96
V9.4	0.51	0.36	0.64	0.73	0.96
V9.5	0.47	0.37	0.64	0.73	0.96
V9.6	0.43	0.38	0.64	0.67	0.96
V9.7	0.44	0.35	0.45	0.60	0.96
V9.8	0.48	0.37	0.69	0.73	0.96
V9.9	0.39	0.41	0.42	0.36	0.97
V9.10	0.52	0.35	0.62	0.64	0.96
V9.11	0.67	0.38	0.44	0.35	0.97
V11.1	0.52	0.35	0.71	0.78	0.96
V11.2	0.50	0.35	0.73	0.78	0.96
V11.3	0.56	0.34	0.62	0.71	0.96

V11.4	0.57	0.33	0.62	0.72	0.96
V11.5	0.56	0.35	0.69	0.76	0.96
V11.6	0.55	0.37	0.64	0.68	0.96
V11.7	0.55	0.36	0.73	0.74	0.96
V11.8	0.55	0.37	0.71	0.79	0.96
...					

Scale	M	SD	D	α_C
Confidence	0.52	0.23	0.56	0.97

2.2.2 Evaluation questionnaires

Prior to performing factor analyses, all data were checked regarding their suitability for this method (Table S6). With a Kaiser-Meyer-Olkin value ≥ 0.6 and a significant Bartlett test for sphericity, the requirements for performing a principal component analysis were met for cognitive load as well as tutor behavior questionnaire (George and Mallery, 2019).

Table S6. Test results for requirements of principal component analysis (Kaiser-Meyer-Olkin criterion KMO; Bartlett test for sphericity: Chi-Squared statistics $\mathcal{X}^2$, degrees of freedom df, significance p).

Questionnaire	KMO	Bartlett test			Total variance explained [%]
		$\mathcal{X}^2$	df	p	
Cognitive load	0.74	522.56	66	< 0.001	75.03
Tutor behavior	0.89	293.91	15	< 0.001	68.61

Via Kaiser criterion (eigenvalue > 1) and based on the screeplot, a principal component analysis with varimax rotation for the items of cognitive load yielded a four-factor solution explaining 75.03% of variance. The items CL4, CL5, CL6, and CL7 (extraneous cognitive load) loaded on the first factor, CL1, CL2, and CL3 (intrinsic cognitive load) loaded on the second factor, CL8, CL9, and CL10 (germane cognitive load) loaded on the third factor, and CL11 and CL12 (effort) loaded on the fourth factor (Table S7). Concerning item analyses of the four scales, except of CL10 ("The task enhanced my understanding of the divergence formulas.") all items had a item-total correlation of $r_{it} \geq 0.56$ and a discrimination index of $D_i \geq 50$. Moreover, the reliability of the germane cognitive load scale was improved from 0.79 to 0.84 by excluding CL10. As CL10 demonstrated the lowest factor loading and the lowest communality, and because CL10 was highly similar to CL9 ("The task enhanced my understanding of divergence."), CL10 was removed from the scale.

A principal component analysis with varimax rotation for tutor behavior revealed, via the Kaiser criterion (eigenvalue > 1) and based on the screeplot, that all items loaded on one factor explaining 68.61% of variance (Table S8). Accordingly, the scale can be considered unidimensional. Since no psychometric abnormalities were found, all items were retained.

Table S7. Results of factor, item, and scale analyses for the cognitive load questionnaire (factors 1, 2, 3, 4, communality κ, item mean M_i, item standard deviation SD_i, item discrimination index D_i, item-total correlation r_{it}, Crombach's alpha when item i is excluded α_{ex}, scale mean M, mean standard deviation SD, mean discrimination index D, Crombachs Alpha α_C, Spearman-Brown coefficient ρ).

	Factor analysis					Item analysis					excl. CL10		
Item	1	2	3	4	κ	M_i	SD_i	D_i	r_{it}	α_{ex}	D_i	r_{it}	α_{ex}
Extraneous cognitive load													
CL6	0.88	0.16	-0.08	-0.03	0.80	0.27	0.28	0.58	0.76	0.72			
CL4	0.78	0.08	-0.18	0.12	0.66	0.33	0.28	0.52	0.56	0.81			
CL5	0.74	0.24	-0.06	-0.25	0.67	0.28	0.29	0.53	0.62	0.79			
CL7	0.69	0.38	-0.13	-0.12	0.64	0.33	0.26	0.50	0.64	0.78			
Intrinsic cognitive load													
CL1	0.22	0.90	0.03	0.11	0.87	0.49	0.28	0.68	0.83	0.78			
CL3	0.14	0.89	-0.02	0.08	0.81	0.47	0.25	0.57	0.76	0.85			
CL2	0.29	0.82	0.06	0.02	0.75	0.40	0.28	0.61	0.74	0.87			
Germane cognitive load													
CL9	-0.10	-0.04	0.90	0.14	0.83	0.56	0.28	0.63	0.72	0.60	0.67	0.72	-
CL8	-0.12	-0.06	0.86	0.12	0.77	0.58	0.25	0.60	0.67	0.67	0.58	0.72	-
CL10	- 0.13	0.15	0.67	0.21	0.54	0.60	0.26	0.43	0.51	0.84	-	-	-
Effort													
CL11	-0.03	0.10	0.16	0.88	0.82	0.67	0.28	0.61	0.67	-			
CL12	-0.11	0.07	0.29	0.86	0.84	0.67	0.27	0.64	0.67	-			

Scale (excl. CL10)	M	SD	D	α_C / ρ
Extraneous cognitive load	0.30	0.22	0.53	0.82
Intrinsic cognitive load	0.45	0.24	0.62	0.88
Germane cognitive load	0.57	0.25	0.63	0.84
Effort	0.67	0.25	0.63	0.80

Table S8. Results of factor, item, and scale analyses for the tutor behavior questionnaire (factor 1, communality κ, item mean M_i, item standard deviation SD_i, item discrimination index D_i, item-total correlation r_{it}, Crombach's alpha when item i is excluded α_{ex}, scale mean M, mean standard deviation SD, mean discrimination index D, α_C = Crombachs Alpha).

	Factor analysis		Item analysis				
Item	1	κ	M_i	SD_i	D_i	r_{it}	α_{ex}
T1	0.85	0.72	0.88	0.14	0.27	0.78	0.86
T2	0.84	0.71	0.91	0.12	0.21	0.74	0.87
T3	0.80	0.63	0.68	0.23	0.51	0.71	0.87
T4	0.85	0.73	0.85	0.20	0.37	0.77	0.85
T5	0.80	0.63	0.81	0.23	0.43	0.70	0.87
T6	0.84	0.70	0.93	0.12	0.20	0.74	0.87

Scale	M	SD	D	α_C
Tutor	0.84	0.14	0.33	0.89

2.3 Normal distribution analyses

The scales formed after item exclusion were subjected to normal distribution analyses (Table S9). If the Kolmogorov-Smirnov test and the Shapiro-Wilk test were significant, skewness and kurtosis were considered. If the absolute values of these parameters are ≤ 2, a normal distribution can be assumed (George and Mallery, 2019).

Table S9. Results of normal distribution analyses (test statistics Kolmogorov-Smirnov test K, test statistics Shapiro-Wilk test W, degrees of freedom df, significance p, skewness s, kurtosis k).

		Kolmogorov-Smirnov test			Shapiro-Wilk test				
Variable	Scale	K	df	p	W	df	p	s	k
Performance	V	0.06	116	0.20	0.99	116	0.54	0.06	-0.31
Confidence	C	0.11	41	0.20	0.97	41	0.23	0.26	-0.74
Cognitive load	ECL	0.19	92	< 0.001	0.90	92	< 0.001	1.21	1.46
	ICL	0.08	92	0.10	0.98	92	0.10	0.15	-0.52
	GCL	0.16	93	< 0.001	0.96	93	0.007	-0.26	-0.46
	E	0.16	93	< 0.001	0.92	93	< 0.001	-0.66	-0.49
Tutor behavior	T	0.15	83	< 0.001	0.88	83	< 0.001	-1.27	1.77

REFERENCES

Baily, C., Bollen, L., Pattie, A., Van Kampen, P., and De Cock, M. (2016). Student thinking about the divergence and curl in mathematics and physics contexts. In *Proceedings of the Physics Education Research Conference 2016, College Park, MD* (American Institute of Physics), 51–54. doi:10.1119/perc.2015.pr.008

Baroffio, A., Kayser, B., Vermeulen, B., Jacquet, J., and Vu, N. V. (1999). Improvement of tutorial skills: an effect of workshops or experience? *Academic Medicine* 74, S75–S77

Bollen, L., Van Kampen, P., and De Cock, M. (2015). Students' difficulties with vector calculus in electrodynamics. *Physical Review Special Topics - Physics Education Research* 11, 020129. doi:10.1103/PhysRevSTPER.11.020129

Bollen, L., van Kampen, P., and De Cock, M. (2018). Development, implementation, and assessment of a guided-inquiry teaching-learning sequence on vector calculus in electrodynamics. *Physical Review Physics Education Research* 14, 020115. doi:10.1103/PhysRevPhysEducRes.14.020115

Ding, L. and Beichner, R. (2009). Approaches to data analysis of multiple-choice questions. *Physical Review Special Topics - Physics Education Research* 5, 020103. doi:10.1103/PhysRevSTPER.5.020103

Dolmans, D. H., Wolfhagen, I., Schmidt, H., and Van der Vleuten, C. (1994). A rating scale for tutor evaluation in a problem-based curriculum: validity and reliability. *Medical Education* 28, 550–558. doi:10.1111/j.1365-2923.1994.tb02735.x

George, D. and Mallery, P. (2019). *IBM SPSS Statistics 25 tep by step: A simple guide and reference* (New York: Routledge)

Hahn, L. and Klein, P. (2022). Kognitive Entlastung durch Zeichenaktivitäten? Eine empirische Untersuchung im Kontext der Vektoranalysis. In *Unsicherheit als Element von*

naturwissenschaftsbezogenen Bildungsprozessen, eds. S. Habig and H. van Vorst (Gesellschaft für Didaktik der Chemie und Physik, virtuelle Jahrestagung 2021), 384–387

Klein, P., Hahn, L., and Kuhn, J. (2021). Einfluss visueller Hilfen und räumlicher Fähigkeiten auf die graphische Interpretation von Vektorfeldern: Eine Eye-Tracking-Untersuchung. *Zeitschrift für Didaktik der Naturwissenschaften* 27, 181–201. doi:10.1007/s40573-021-00133-2

Klein, P., Viiri, J., and Kuhn, J. (2019). Visual cues improve students' understanding of divergence and curl: Evidence from eye movements during reading and problem solving. *Physical Review Physics Education Research* 15, 010126. doi:10.1103/PhysRevPhysEducRes.15.010126

Klein, P., Viiri, J., Mozaffari, S., Dengel, A., and Kuhn, J. (2018). Instruction-based clinical eye-tracking study on the visual interpretation of divergence: How do students look at vector field plots? *Physical Review Physics Education Research* 14, 010116. doi:10.1103/PhysRevPhysEducRes.14.010116

Klepsch, M., Schmitz, F., and Seufert, T. (2017). Development and validation of two instruments measuring intrinsic, extraneous, and germane cognitive load. *Frontiers in Psychology* 8, 1997. doi:10.3389/fpsyg.2017.01997

Krell, M. (2017). Evaluating an instrument to measure mental load and mental effort considering different sources of validity evidence. *Cogent Education* 4, 1280256. doi:10.1080/2331186X.2017.1280256

Leppink, J., Paas, F., Van der Vleuten, C. P., Van Gog, T., and Van Merriënboer, J. J. (2013). Development of an instrument for measuring different types of cognitive load. *Behavior Research Methods* 45, 1058–1072. doi:0.3758/s13428-013-0334-1

Scott, T. F., Schumayer, D., and Gray, A. R. (2012). Exploratory factor analysis of a Force Concept Inventory data set. *Physical Review Special Topics - Physics Education Research* 8, 020105. doi:10.1103/PhysRevSTPER.8.020105

Tabachnick, B. G. and Fidell, L. S. (2019). *Using multivariate statistics* (Boston: Pearson)
